# Fast In Vitro Release and In Vivo Absorption of an Anti-Schizophrenic Drug Paliperidone from Its Soluplus^®^/TPGS Mixed Micelles

**DOI:** 10.3390/pharmaceutics14050889

**Published:** 2022-04-19

**Authors:** Ye Zhou, Chenhui Wang, Wenqian Liu, Meiqing Yang, Bohui Xu, Yong Chen

**Affiliations:** School of Pharmacy, Nantong University, 19 Qixiu Road, Nantong 226001, China; 1823310015@stmail.ntu.edu.cn (Y.Z.); 2119310006@stmail.ntu.edu.cn (C.W.); 2023110134@stmail.ntu.edu.cn (W.L.); 2023110146@stmail.ntu.edu.cn (M.Y.); xuzi_2001@ntu.edu.cn (B.X.)

**Keywords:** paliperidone, acute schizophrenia, Soluplus^®^, TPGS, micelles, pharmacokinetics

## Abstract

The purpose of this study was to develop a drug delivery system for paliperidone (PPD) in order to provide a more effective therapeutic strategy for patients with acute schizophrenia. PPD-loaded Soluplus^®^/TPGS mixed micelles (PPD-S/T-MM) were prepared using the thin-film hydration method. The critical micelle concentration (CMC) of blank S/T-MM was 4.77 × 10^−2^ mg/mL. PPD presented much higher solubility in PPD-S/T-MM formulation than that in pure water. The particle size of blank or drug loaded S/T-MM was around 60 nm. The polydispersity index (PDI) was less than 0.1. PPD-S/T-MM presented a nearly spherical shape under transmission electron microscopy. The encapsulation efficiency (EE%) of PPD-S/T-MM was higher than 94%. Based on the analysis of XRD and DSC, it was proved that PPD was incorporated in the core of the mixed micelles as amorphous dispersion or solid solution. PPD-S/T-MM were stable when they were undergoing dilution with water and the change of environmental pH. Although PPD-S/T-MM showed lower rates to release PPD than those from PPD raw material in acidic solution, they provided faster release rates in neutral conditions than those from PPD raw material who only showed modest dissolution in the same neutral condition. This proves that PPD-S/T-MM can release PPD in a more controlled manner. After oral administration of PPD-S/T-MM (dose of PPD, 6 mg/kg) in rats, the plasma concentration of PPD increased rapidly: T_max_ was 0.83 ± 0.29 h, and C_max_ was 844.33 ± 93.73 ng/mL. Oral administration of PPD suspension resulted in longer T_max_ and lower C_max_. The relative oral bioavailability was about 158% for PPD-S/T-MM over PPD suspension. These findings confirm that PPD-S/T-MM can provide faster release in neutral conditions and better oral absorption in rats than those from PPD raw material, which should potentially benefit patients with acute schizophrenia.

## 1. Introduction

Schizophrenia is characterized by significant impairments in the way reality is perceived and is associated with a considerable disability that may affect all areas of life including personal, family, social, educational, and occupational functioning [1]. This mental disease affects approximately 24 million people or 1 in 300 people (0.32%) worldwide [1]. The prevalence rate of this disease can reach 6.55‰ in China [2]. Acute schizophrenia is a kind of schizophrenia with acute onset and short course, which is mainly characterized by positive symptoms, such as delusions, hallucinations, behavior disorders and agitation [3]. Patients with acute schizophrenia are often destructive and dangerous to themselves and others [4]. At present, a few atypical antipsychotics are clinically used to treat schizophrenia given to better therapeutic efficacies to manage positive symptoms and improve the cognitive function of the patients with schizophrenia. Moreover, it has less incidence of extrapyramidal symptoms and endocrine related adverse events [5].

**Figure 1:**
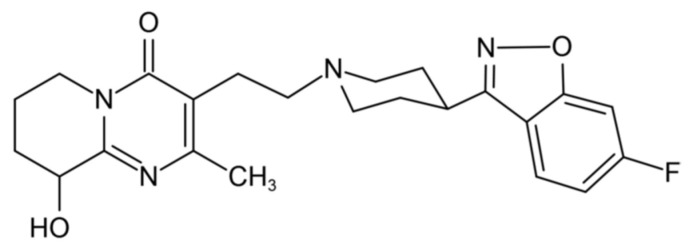
The chemical structure of paliperidone (PPD; M.W. 426.48, Log P 3.0, pKa_1_ = 8.2, piperidine moiety; pKa_2_ = 2.6, pyrimidine moiety [12]).

Paliperidone (PPD, Figure 1) is one of the atypical antipsychotics, which is also known as 9-hydroxyrisperidone—an active metabolite of risperidone. PPD rebalances dopamine and serotonin to improve thinking, mood, and behavior hence it has been used clinically to treat schizophrenia and schizoaffective disorder. To date, marketed PPD formulations are extended-release tablets and long-acting injectables (Table 1). These formulations successfully address the issue of noncompliance with long-term maintenance treatment in schizophrenic patients by reducing the frequency of the drug administration on one hand; however, they could also encounter limitations in the acute setting or where patients have adherence issues on the other hand. There is no fast release formulation of PPD marketed. Meanwhile, fast release formulations, such as orally-disintegrating tablets of risperidone and olanzapine, and orally-disintegrating film of loxapine have been clinically used to address acute schizophrenia. The technologies to develop fast release formulations of antipsychotics have been reported elsewhere in recent years. Besides the abovementioned orally-disintegrating tablets or films, for example, engineered nanoparticles prepared by the acid-alkali neutralization method can significantly enhance the dissolution rate of risperidone [6]. Moreover, nanocrystals of aripiprazole incorporated into buccoadhesive chitosan films could also provide a rapid drug delivery to the systemic circulation [7]. Intranasal administration of PPD nanoemulsion and Tween 80—based micelles was reported to reduce the locomotor activity of mice more rapidly than that after administration of PPD suspension [8].

Polymeric micelles are formed by self-assembly of amphiphilic polymers, with a hydrophobic core as a reservoir for lipophilic drugs. Depending on the physicochemical properties of the drug and those of the polymer chains forming the micellar structure, the core of polymeric micelles can solubilize considerable amounts of hydrophobic drug molecules which otherwise would precipitate in the gastrointestinal fluids. Nevertheless, polymeric micelles formed from single copolymers often lack stability, exhibit unsatisfactory drug loading capacity, or have broad size distribution, primarily due to limitations in the number of available building blocks [9]. Conversely, the mixed micellar nanocarriers offer significant improvements with regard to carrier stability and improved drug encapsulation efficacy [10]. Therefore, they have been widely investigated to enhance dissolution rates and oral bioavailability of poorly water-soluble drugs [11]. 

PPD presented moderate absolute oral bioavailability in female (78 %) and male (46%) rats [12]; however, its bioavailability in humans is much poorer (~23%) [13]. Its water solubility was as low as 0.003% (*w*/*v*) [12], and it is classified as a BCS II drug [14]. The aim of this study was to develop mixed micelles based on two biocompatible copolymers of polyvinyl caprolactam–polyvinyl acetate–polyethylene glycol (Soluplus^®^) and D-α-tocopheryl polyethylene-glycol 1000 succinate (TPGS), to improve the aqueous solubility, dissolution rates and oral bioavailability of PPD. 

## 2. Materials and Methods

### 2.1. Materials

PPD raw materials (purity ˃ 98.0%) were purchased from Widely Chemicals (Wuhan, China). Soluplus^®^ (batch No. 66458088Q0) was kindly gifted from BASF (Ludwigshafen, Germany). D-α-Tocopherol polyethylene glycol 1000 succinate (TPGS) was purchased from Hongxin Ruiyu Fine Chemicals (Wuhan, China). HPLC-grade methanol was supplied by Merck KGaA (Darmstadt, Germany). All aqueous solutions were prepared using Milli-Q^®^ water (resistivity ˃ 18 MΩ·cm). All other chemicals used were at least of analytical grade.

### 2.2. Animals

Male Sprague-Dawley (SD) rats (B.W. 220 ± 20 g) were supplied by the Laboratory Animal Center of Nantong University. All the animals were in a 12-h dark-light cycle animal facility with controlled temperature and humidity and had free access to regular chow and water except for test sessions. The study was approved by the Laboratory Animal Ethics Committee of Nantong University. All animal experimental protocols were performed according to the NIH (National Institutes of Health USA) (2011) Guide for the Care and Use of Laboratory Animals (protocol code S20210317-501).

### 2.3. Preparation of the Mixed Micelles

**Figure 2:**
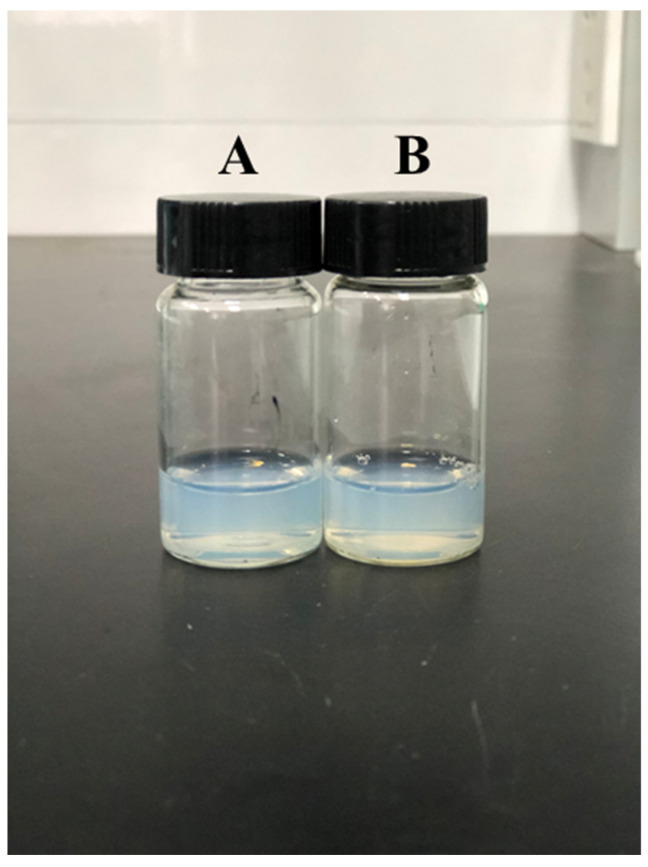
Appearance of blank S/T-MM (**A**) and PPD-S/T-MM-2.0 (**B**).

The mixed micelles of PPD were prepared by the thin-film hydration method [15,16,17]. Briefly, Soluplus^®^ (230 mg) and PPD (2 mg, 5 mg or 10 mg) were added into a flask (50 mL) and dissolved in acetone (10 mL) with the aid of an ultrasonic bath, followed by evaporation of acetone using a rotary evaporator to yield a thin film. Then, TPGS (19.2 mg) was dissolved in water (5 mL) to form an aqueous solution and subsequently added to the flask with the thin film. The materials were spontaneously assembled into micelles upon magnetic stirring. Finally, slightly opalescent PPD-loaded Soluplus^®^/TPGS mixed micellar formulations (PPD-S/T-MM) with the theoretical PPD concentrations at 0.4 (PPD-S/T-MM-0.4), 1.0 (PPD-S/T-MM-1.0) and 2.0 (PPD-S/T-MM-2.0) mg/mL were obtained (Figure 2). Blank S/T-MM were prepared using the same procedure without the addition of PPD. To achieve lyophilized products, 5 mL of blank S/T-MM, PPD-S/T-MM-0.4, PPD-S/T-MM-1.0 and PPD-S/T-MM-2.0 were subjected to initial freezing (−20 °C for 2 h), deep freezing (−80 °C for 12 h) and lyophilizing (−40 °C for 24 h at 5 Pa) to yield white lyophilizates accordingly.

### 2.4. Characterization of the Mixed Micelles

#### 2.4.1. Critical Micelle Concentration (CMC)

CMC was determined by ultraviolet-visible (UV-Vis) spectroscopy [17]. Briefly, the KI/I_2_ stock solution was prepared by dissolving 0.5 g of iodine (I_2_) and 1 g of potassium iodide (KI) in 50 mL of water. The lyophilized blank S/T-MM sample was dissolved in water and the sample was further diluted into a series of working solutions with polymer concentrations varied from 0.01 μg/mL to 0.5 mg/mL; 25 μL of KI/I_2_ working solution was added to micellar formulations. Prior to measurement, the mixtures were incubated in dark for 12 h at room temperature. The absorbance was measured at 366 nm for three times. The absorbance was plotted against the logarithm of the polymer concentration. The CMC value corresponded to the concentration of the polymer where the absorbance dramatically increased.

#### 2.4.2. Particle Size, Polydispersity Index (PDI) and Zeta Potential Analysis

The particle size, PDI and zeta potential of the mixed micelles were determined by a particle size analyzer (NanoBrook 90Plus, Brookhaven Instruments; Holtsville, NY, USA). Blank S/T-MM, PPD-S/T-MM-0.4, PPD-S/T-MM-1.0 and PPD-S/T-MM-2.0, obtained by dissolving corresponding lyophilizates into 5 mL of water, were diluted 5-fold with water before the measurement. The measurement was performed in triplicate at 25 °C. 

#### 2.4.3. Encapsulation Efficiency and Drug Loading Efficiency

An aliquot (1 mL) from blank S/T-MM, PPD-S/T-MM-0.4, PPD-S/T-MM-1.0, or PPD-S/T-MM-2.0, obtained by dissolving corresponding lyophilizates into 5 mL of water, was placed into a 2 mL of EP tube and centrifuged at 4000 r/min for 10 min [17]. Then, an aliquot of the supernatant (0.5 mL) was added to a 10 mL volumetric flask, diluted with methanol, and treated with a KQ-500VDE ultrasonic cleaner (Shumei Ultrasonic Instruments; Kunshan, China) for 20 min to release the encapsulated PPD. The concentration of PPD in the obtained solution was determined using HPLC-UV without further dilution (see Section 2.8). From the concentration of PPD determined, the weight of PPD encapsulated in the micelles can be determined. The encapsulation efficiency (EE%) and drug loading efficiency (DL%) can be calculated using the following equations. The experiments were performed in triplicates.

$$\mathrm{EE}\%=\frac{\mathrm{weight}\;\mathrm{of}\;\mathrm{drug}\;\mathrm{in}\;\mathrm{micelles}}{\mathrm{weight}\;\mathrm{of}\;\mathrm{drug}\;\mathrm{added}}\times 100\%$$

$$\mathrm{DL}\%=\frac{\mathrm{weight}\;\mathrm{of}\;\mathrm{drug}\;\mathrm{in}\;\mathrm{micelles}}{\begin{array}{c} \mathrm{weight}\;\mathrm{of}\;\mathrm{excipients}\;\mathrm{added}\\ +\mathrm{weight}\;\mathrm{of}\;\mathrm{drug}\;\mathrm{added} \end{array}}\times 100\%$$

#### 2.4.4. Differential Scanning Calorimetry (DSC)

Appropriate amounts of PPD raw materials, Soluplus^®^, TPGS, physical mixture (PPD, Soluplus^®^ and TPGS mixed physically within the same proportions as preparing PPD-S/T-MM-2.0), and the lyophilizates of PPD-S/T-MM-2.0 were subjected to DSC analysis (DSC 8000, PerkinElmer, Shelton, CT, USA), respectively. Each sample was sealed in an aluminum plate and heated from 25 °C to 300 °C at a heating rate of 10 °C /min [18].

#### 2.4.5. X-ray Diffraction (XRD)

Appropriate amounts of PPD raw materials, Soluplus^®^, TPGS, physical mixture (PPD, Soluplus^®^ and TPGS mixed physically within the same proportions as preparing PPD-S/T-MM-2.0), and the lyophilizates of PPD-S/T-MM-2.0 were subjected to XRD analysis (SmartLab SE, Rigaku, Tokyo, Japan), respectively. The measuring voltage was 40 kV, and the current was 25 mA. The scanning was carried out at a rate of 0.9°/min within the scanning angle ranged from 5° to 40° [18].

#### 2.4.6. Transmission Electron Microscopy (TEM)

A drop of sample from PPD-S/T-MM-2.0, obtained by dissolving its lyophilizates into 5 mL of water, was placed on a copper mesh and stained with 2% (*w*/*v*) phosphotungstic acid solution. After the sample was dried at room temperature, its morphology was observed under TEM (JEM-2100, JEOL Ltd., Tokyo, Japan) using an acceleration voltage of 120.0 kV [19].

### 2.5. Stability Studies

#### 2.5.1. Dilution Stability

PPD-S/T-MM-1.0 or PPD-S/T-MM-2.0, obtained by dissolving corresponding lyophilizates into 5 mL of water, were diluted 5, 50 or 100-fold with physiological saline, respectively. The particle size and PDI were measured. The dilution stability study was carried out in triplicates.

#### 2.5.2. pH Stability

Simulated gastric fluid (SGF, pH 1.0) and simulated intestinal fluid (SIF, pH 6.8) was prepared: 1 L of SGF was composed of 0.2% w/w NaCl and 0.0825 mol/L HCl, and the pH value was adjusted to 1.0 [20]; 1 L of SIF was composed of 6.805 g of NaH_2_PO_4_ and 0.896 g of NaOH, and the pH value was adjusted to 6.8 [21]. Enzymes were not added in order to avoid the influence of their catalytic activity on the micellar components; 0.5 mL of sample from PPD-S/T-MM-1.0 or PPD-S/T-MM-2.0, obtained by dissolving corresponding lyophilizates into 5 mL of water, was diluted to 10 mL with SGF or SIF, respectively. The samples were placed in a 37 °C water bath and stirred at 120 r/min [22]. Samples were taken at 0, 2, and 4 h to determine whether the particle size changed. The pH stability study was carried out in triplicates.

### 2.6. In Vitro Drug Release

Two milliliters of sample from PPD-S/T-MM-1.0 or PPD-S/T-MM-2.0, obtained by dissolving corresponding lyophilizates into 5 mL of water, were placed into a dialysis bag (MWCO = 3500 Da), respectively [23]. Then, the dialysis bag was immersed into a release medium (200 mL) under magnetic stirring (100 r/min) [15]. SGF (pH 1.0) and PBS (pH 7.4) containing 0.5% Tween 80 (*w*/*v*) were used as the release media, respectively; 1 mL of aliquot was taken from the release media at the predetermined time intervals (15, 30, 60, 120, 180, 240, 360 and 480 min). After sampling, 1 mL of fresh release media solution was supplemented. The PPD raw materials were used as a control, which was prepared by suspending 2.0 or 4.0 mg of PPD raw materials in 2 mL of water, respectively. The release profiles were then investigated in the same way. All samples were measured by HPLC-UV (see Section 2.8), and the percentages of the cumulative release at different time points were calculated. The experiments were performed in triplicate.

### 2.7. Pharmacokinetic Study

SD rats were fasted for 12 h with free access to water before the experiment. CMC-Na (0.5 g) was dissolved in water (100 mL) to prepare a 0.5% CMC-Na solution. An appropriate amount of PPD raw materials was added to the solution and was magnetically stirred overnight to obtain PPD suspension with the final PPD concentration at 2 mg/mL. PPD-S/T-MM-2.0 was obtained by dissolving its lyophilizates into 5 mL of water right before the oral administration. SD rats were randomly divided into two groups (*n* = 3), and each group was administered intragastrically with either PPD suspension or PPD-S/T-MM-2.0 at a dose of 6 mg/kg [24], respectively. Blood samples withdrawn from the orbital venous plexus were collected into heparinized tubes at 0.25, 0.5, 1, 2, 3, 4, 6, 8, 12 and 24 h. The samples were immediately centrifuged at 10,000 r/min for 10 min to separate the plasma. The plasma was stored in a refrigerator at −20 °C before further analysis. 

Prior to analysis, the plasma samples were thawed at room temperature; 100 μL of plasma sample was mixed with 50 μL of internal standard solution (risperidone, RIS, 100 ng/mL, in methanol) and 100 μL of acetonitrile. The mixtures were vortexed to mix evenly, then centrifuged for 10 min at 4 °C at 15,000 r/min; 100 μL of the supernatant was subjected to analysis (see Section 2.8).

### 2.8. Quantification of PPD

Quantification of PPD concentrations in non-plasma samples was carried out on a high-performance liquid chromatography, equipped with an LC-10AT VP pump and an SPD-10A VP ultraviolet-visible detector (HPLC-UV, Shimadzu Corporation; Kyoto, Japan). Isocratic separation was performed using a 150 × 4.6 mm Diamonsil^®^ column packed with 5 μm C18 end-capped silica reversed-phase particles. The mobile phase consisted of methanol/phosphate buffer (20 mM H_3_PO_4_; pH 3.1) (40/60, *v*/*v*) with a flow rate of 1.0 mL/min. The column temperature was kept at 30 °C, and the injection volume was 20 μL. The detection wavelength was set at 237nm [25]. Data were collected and processed by Labsolution workstation. The method for quantification of PPD was linear over a range of 2~200.0 μg/mL. The method was validated with three replicates at 5, 50 and 200 μg/mL and showed good precision and accuracy for both intra-and inter-day analyses. The limit of detection (LOD) of PPD, defined as three times over the baseline noise, was 25 ng/mL. The limit of quantitation (LOQ) of PPD, defined as 10 times over the baseline noise, was 50 ng/mL. 

Quantification of PPD plasma concentrations was performed on an Acquity^®^ ultra-performance liquid chromatography coupled with Quattro Premier XE tandem mass spectrometer (UPLC-MS/MS, Waters Corporation, Milford, CT, USA). The chromatographic separation was achieved using an Acquity^®^ UPLC BEH C18 column (50 mm × 2.1 mm, 1.7 μm). The mobile phase consisted of solvent (A, methanol) and solvent (B, 0.01 mol/L ammonium formate solution, pH 3.5). The following parameters for gradient elution were used: the percentage of solvent B decreased linearly from 70% to 10% over the first 1.5 min; then held at 10% for 1 min; then increased to 70% linearly between 2.5 and 2.6 min; then hold on at 70% until 4 min. The flow rate of the mobile phase was 0.2 mL/min. The column temperature was kept at 40 °C and the injection volume was 5 μL. For the mass spectrometry detection, electrospray ionization was set at a positive-ion mode (ESI+), and the multiple reaction monitoring mode (MRM) was employed for quantitative analysis with the parent-daughter ion transitions of m/z 427.45→207.18 for PPD and m/z 411.42→191.19 for IS (risperidone, RIS). The capillary voltage was 3.5 kV; ion source temperature was 180 °C; nitrogen was used as the nebulizer gas and auxiliary gas and the flow rates were 450 L/h and 50 L/h, respectively; argon was used as collision gas and the flow rate was 0.2 mL/min. Data processing was performed using Waters MassLynx software. A stock solution of PPD (1 mg/mL) was prepared by dissolving appropriate amount of PPD into methanol. The stock solution was further diluted to obtain a series of working solutions with PPD concentrations of 10,000, 5000, 1000, 500, 100, 50 and 10 ng/mL. 10 μL of each working solution was mixed with 100 μL of blank plasma to obtain a series of standard solutions with the final PPD concentrations of 1000, 500, 100, 50, 10, 5 and 1 ng/mL, respectively. The working solution of internal standard RIS (100 ng/mL) was prepared in methanol as well. The method for quantification of PPD was linear over a range of 1~1000 ng/mL. The method was validated with three replicates at 1, 10, 50 and 500 ng/mL and showed good precision and accuracy for both intra-and inter-day analyses. The LOQ for PPD was 1 ng/mL. 

### 2.9. Statistical Analysis

Data are from at least three independent experiments and all values are expressed as mean ± standard deviation. The Student’s *t*-test was used for statistical comparison. A significance value of * *p* < 0.05 was determined to be significant in all cases.

## 3. Results and Discussion

### 3.1. CMC Determination

CMC is a critical indicator of micellar stability and micellization ability: the lower the CMC value, the easier it is to prepare micelles and the more stable they will be. Currently, there are various methods, such as tensiometry, conductometry, fluorimetry, calorimetry, light scattering, and nuclear magnetic resonance (NMR) spectroscopy for the determination of the CMC [26].

**Figure 3:**
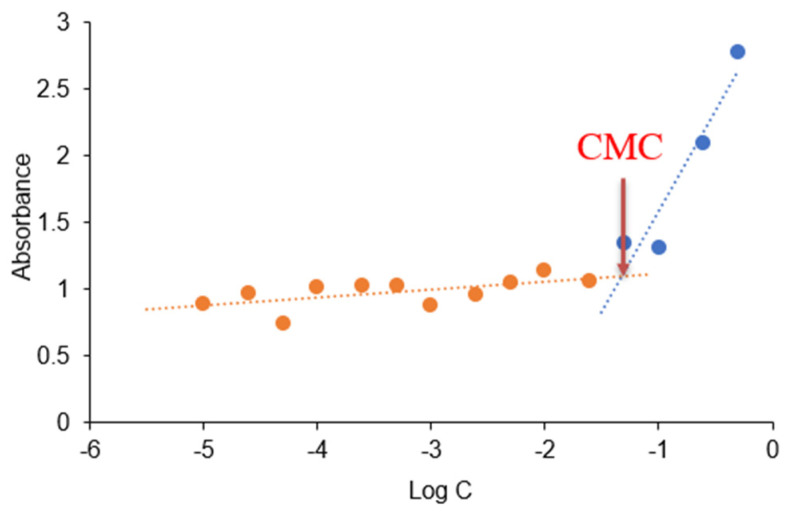
Plots of ultraviolet absorbance of I_2_ versus concentration of the mixed micelles in water. CMC value was calculated by corresponding polymer concentration where a sharp increase in absorbance is observed. The orange and blue dots represent the polymer concentrations before and after the sharp increase in absorbance, respectively. The crosspoint between the orange and blue curves represents the CMC. (Mean ± SD, *n* = 3).

In this study, ultraviolet spectrometry was used to measure the CMC value of the mixed micelles. The scatter plots shown in Figure 3 were obtained from the experiment, and the fitted linear curves were drawn. The CMC value of the mixed micelle can be determined from the intersection of the two fitted curves. The determined CMC value was 4.77 × 10^−2^ mg/mL, which was higher than the CMC value of pure Soluplus^®^ (7.6 × 10^−3^ mg/mL), given by the manufacturer [27] without further specification of method, solvent or temperature. The increase in the CMC could be attributed to the addition of TPGS which presents a much higher CMC value (0.2 mg/mL) [15]—the incorporation of TPGS into Soluplus^®^ micelles has a negative impact on the Soluplus^®^ self-aggregation [28]. However, different methods and conditions applied to determine the CMC would also affect the value in many ways. For example, the CMC, determined by an isothermal titration calorimetry method, was estimated to be approximately 0.8 mg/mL for Soluplus^®^ at 25 °C [29]. Another report gave the CMC value of Soluplus^®^/TPGS (molar ratio 6:1) mixed micelles at about 0.016 mg/mL [17]. Nevertheless, the CMC value in our study suggested that the mixed micelle could still remain the integrity upon dilution since a CMC value less than 135 mg/L is considered enough to resist dissociation upon dilution after oral administration [30].

### 3.2. Particle Size, PDI and Zeta Potential Analysis

In this study, the particle size, PDI and zeta potential of the drug loaded micelles were measured by dynamic light scattering.

**Figure 4:**
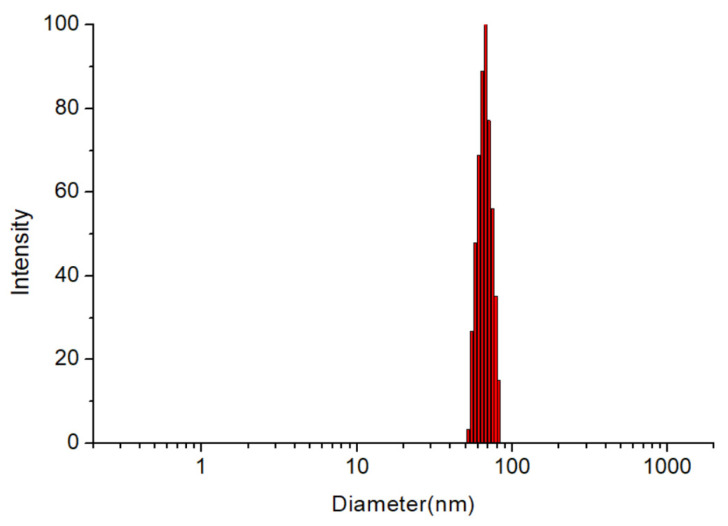
Particle size distribution in PPD-S/T-MM-1.0.

As shown in Table 2 and Figure 4, the particle size of blank S/T-MM was 61.3 ± 0.3 nm. Incorporation of PPD into the mixed micelles using the thin-film hydration method did not change the particle size significantly: the particle sizes of PPD-S/T-MM-0.4, PPD-S/T-MM-1.0 and PPD-S/T-MM-2.0 were about 60 nm as well. In general, micelles with a particle size of <100 nm would benefit from oral intestinal absorption [31], and the particle size of Soluplus^®^-based micelles is around 60~70 nm [32,33,34]. The PDI values of the micelles were less than 0.1, suggesting that the mixed micelles exhibited a narrow and unimodal size distribution. The measured zeta potential ranged from −1.65 mV to –4.57 mV, which was very close to many previous reports [19,32,33]; however, it was also found that the increase in drug loading led to an increase in the absolute value of zeta potential. This needs to be explained in further investigations. The saturated solubility of PPD in distilled water is 0.033 mg/mL [35], and the mixed micelles can dissolve as much as 2 mg/mL of PPD—the solubility is enhanced by at least 60 times in this study.

### 3.3. Encapsulation Efficiency and Drug Loading Capacity

The mass ratio of Soluplus^®^ and TPGS to prepare the mixed micelles was about 12:1. The high proportion of Soluplus^®^ used was to maintain a low CMC value [15] by which the high encapsulation efficiency could be achieved. Table 3 shows DL% and EE% of drug loaded micelles at three PPD concentrations. Using the thin-film hydration method, PPD was successfully incorporated into S/T-MM with a high EE% of more than 94%. The theoretical DL% of drug-loaded S/T-MM with PPD concentrations of 0.4, 1, and 2 mg/mL were 0.80%, 1.97%, and 3.86%, respectively, which were close to the determined DL%—the difference was less than 0.17%, indicating again that the added PPD was incorporated into the S/T-MM with high efficiency. The drug loading efficiency was not intended to increase further given the low daily dose needed clinically (1.5 mg~9 mg, as shown in Table 1).

### 3.4. DSC

**Figure 5:**
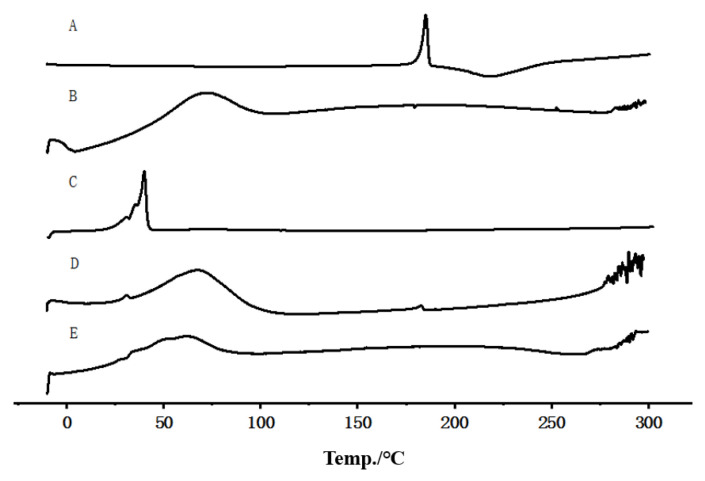
DSC thermograms of PPD (**A**), Soluplus^®^ (**B**), TPGS (**C**), physical mixture (**D**) and lyophilizates of PPD-S/T-MM-2.0 (**E**).

The DSC thermograms of PPD raw material, Soluplus^®^, TPGS, the physical mixture, and the lyophilizates of PPD-S/T-MM-2.0 are presented in Figure 5. The endothermic melting peak of PPD is at 184.83 °C (Figure 5A), which was close to a previous report where the endothermic peak of PPD was found at 192 °C [36]. It was observed that Soluplus^®^ (Figure 5B) presented a very broad melting peak in the range from 50 °C to 100 °C which corresponded to the melting process of this polymer with a *T*g of approximately 70 °C [37], whereas TPGS (Figure 5C) had a melting peak at 34.89 °C, which was close to a previous report [38]. The corresponding endothermic peaks of PPD, Soluplus^®^ and TPGS can be observed in the thermogram of the physical mixture (Figure 5D). The endothermic peak of PPD disappeared in the DSC thermogram of the lyophilizates of PPD-S/T-MM-2.0 (Figure 5E), indicating that PPD was completely entrapped within the mixed micelles as an amorphous dispersion or solid solution in the polymer matrix.

### 3.5. XRD

**Figure 6:**
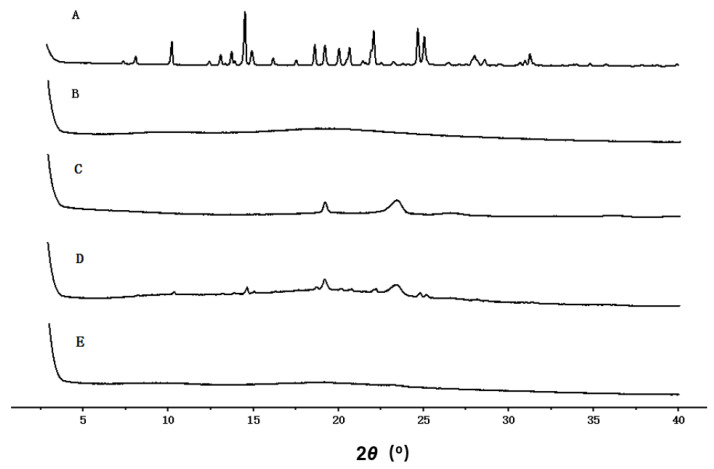
XRD spectrum of PPD (**A**), Soluplus^®^ (**B**), TPGS (**C**), physical mixture (**D**) and lyophilizates of PPD-S/T-MM-2.0 (**E**).

The diffraction patterns of the samples are shown in Figure 6. It can be seen from Figure 6A that PPD had sharp crystal diffraction peaks at 10.24°, 14.54°, 14.94°, 18.64°, 19.16°, 21.98°, 24.62°, 25.0°, 28.54° and 31.16°. Figure 6B shows that Soluplus^®^ presented a very broad and low diffraction peak. Figure 6C shows that TPGS presented two diffraction peaks at 19.04° and 23.37°, which weresimilar to a previous report [38]. In Figure 6D, a diffraction peak is observed at 14.94°, which was the representative diffraction peak of PPD, and the two diffraction peaks of TPGS can be clearly observed as well. There were no sharp diffraction peaks observed in the lyophilizates of PPD-S/T-MM-2.0 (Figure 6E), indicating again that the entrapped PPD in the mixed micelles was in a disordered crystalline or amorphous state. The intensified interaction between PPD with the hydrophobic core of the mixed micelles led to the disappearance of the crystalline order of PPD [38]. 

### 3.6. TEM

**Figure 7:**
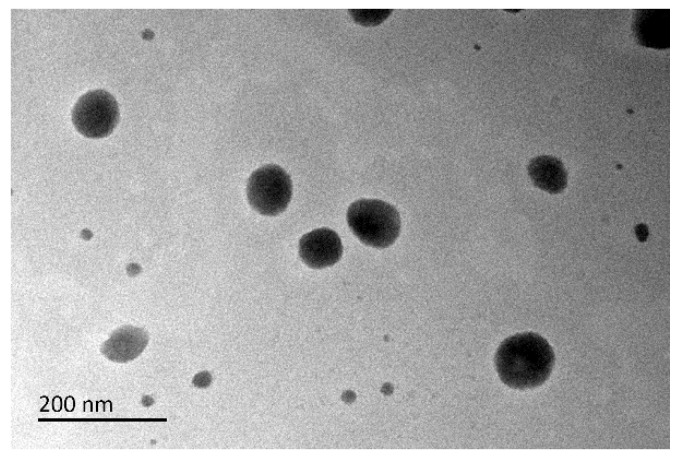
TEM image of PPD-S/T-MM-2.0.

The mixed micelles presented a nearly spherical shape under TEM observation (Figure 7). The particle size of the micelles was about 60 nm, which was close to the size measured by the particle size analyzer (see Table 2).

### 3.7. Stability Studies

#### 3.7.1. Dilution Stability

The dilution stability of mixed micelles is crucial for successful drug delivery. Once the aggregative micelles break apart due to dilution after oral administration, the encapsulated hydrophobic drug releases before reaching the area of absorption, resulting in unsuccessful enhancement of the oral bioavailability. In this study, the particle size and PDI of the PPD-S/T-MM did not change significantly even after undergoing a 100-fold dilution (Table 4), which should be attributed to the low CMC of the mixed micelles—it allowed the micelles to remain relatively stable at low polymer concentrations. This indicates that PPD-S/T-MM should have acceptable ability against dilution after oral administration.

#### 3.7.2. pH Stability

The data in Table 5 shows that the particle size of PPD-S/T-MM in SGF and SIF did not change markedly during the experiments, indicating that PPD-S/T-MM can maintain the structural integrity in a very acidic gastrointestinal environment. This may be due to the protective effect of the PEG chain on the micellar surface against the change in environmental pH value [15].

### 3.8. In Vitro Drug Release

**Figure 8:**
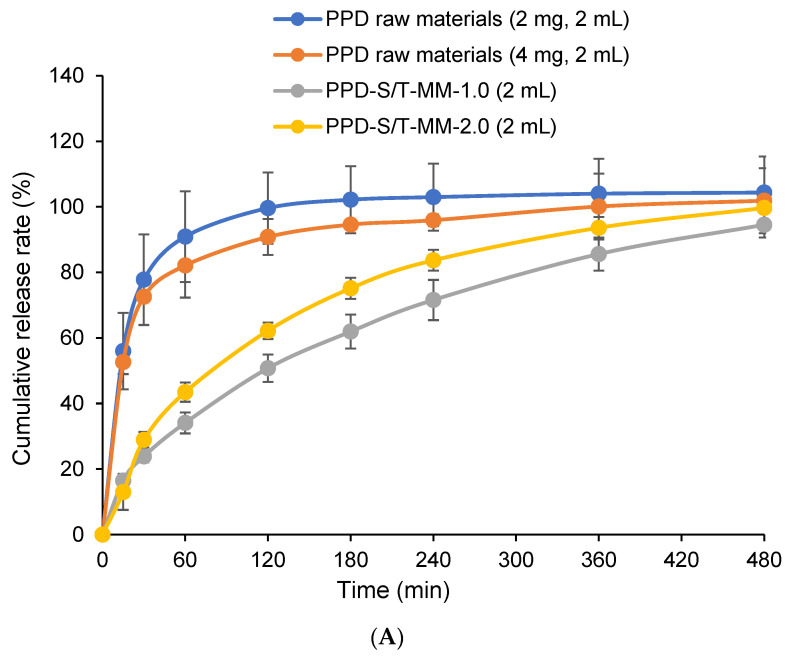

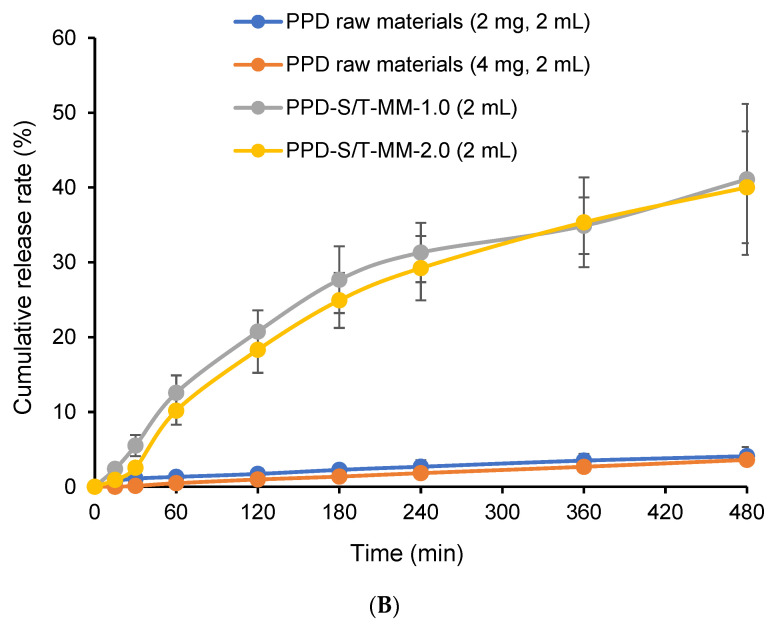
In vitro release of PPD (mean ± SD, *n* = 3). Release of PPD from PPD raw materials and PPD-S/T-MM in SGF (**A**); and release of PPD from PPD raw materials and PPD-S/T-MM in PBS containing 0.5% (*w*/*v*) Tween 80 (**B**).

The release of PPD from the raw material and from the PPD-S/T-MM are expressed in terms of the percentage of PPD released plotted versus time (Figure 8).

In SGF (Figure 8A), the cumulative release of PPD from PPD-S/T-MM-1.0 and PPD-S/T-MM-2.0 within 8 h was 94.55 ± 3.93% and 99.67 ± 3.99%, respectively, and the cumulative release of PPD from its raw materials (2 mg and 4 mg) within the same time reached 104.36 ± 10.99% and 101.88 ± 9.94%—they were comparable. However, if the cumulative release of PPD was compared within 0 to 3 h, the differences between PPD raw materials and PPD-S/T-MM were significant—the latter formulations presented slower release rates than the former formulations, indicating that the dissolution of PPD from its raw materials in SGF is rapid. This could be attributed to the high solubility of PPD in acidic solutions [12]. The slow release of PPD from PPD-S/T-MM proves that a strong hydrophobic interaction between PPD and the inner core of the mixed micelles does exist; furthermore, it also reveals that the micelles are relatively stable in acidic conditions. In a PBS solution (pH 7.4) containing 0.5% (*w*/*v*) Tween 80 (Figure 8B), the sink condition can be guaranteed since the solubility of PPD was determined as 0.72 ± 0.08 mg/mL in this solution, which is much higher than the theoretical concentration of PPD even if the total 4 mg of PPD was completely dissolved within the 200 mL of the dissolution media. In this case, the cumulative release of PPD from PPD-S/T-MM-1.0 and PPD-S/T-MM-2.0 within 8 h were 41.10 ± 10.10% and 40.04 ± 7.47%, respectively, and PPD raw materials showed very low levels of release percentages (<5%). Although both SGF and PBS (pH 7.4) containing 0.5% Tween 80 (*w*/*v*) can provide sink conditions, the release rates of PPD from its raw materials were completely different. Given the Noyes–Whitney equation, the dissolution rate of the solute depends on the surface area of the solute particle, the diffusion coefficient, the thickness of the concentration gradient, the solute concentration at the particle surface (saturation), and the solute concentration in the bulk solvent/solution [39]. If the solute presents different saturated concentrations at the particle surface in different solvents, such as PPD which presents much higher saturated solubility in an acidic environment than that in a neutral environment, the dissolute rates of the solute should be different in these different solvents as well. This explains the reason that PPD is released more rapidly from its raw materials in the acidic SGF than that in the neutral PBS solution. It is also worthwhile to note that the dissolution rates and extents of PPD from PPD-S/T-MM in SGF and PBS solutions were very different—PPD was apparently released more rapidly and completely in the former condition. This could be attributed to the fact the change of pH would slightly impact the microstructure of the micelle [40], and the changes of surface charges of PPD molecules in different dissolution media could affect the hydrophobic interaction between PPD and the hydrophobic core of the mixed micelles—PPD could be partially ionized at the very acidic SGF but not in at the neutral PBS solution given to the zwitterionic property (pKa_1_ = 8.2, piperidine moiety; pKa_2_ = 2.6, pyrimidine moiety) [12]. These results would also suggest that after oral administration of unencapsulated PPD, PPD may dissolve in the stomach due to the acidic gastric juice; however, the dissolved molecules would crystalize again after going through the intestinal tract with a weakly acidic or nearly neutral environment hence the absorption could be hindered due to the intestinal barrier to transport the recrystallized nanoparticles. In contrast, appreciable amounts of encapsulated PPD could remain in the core of the mixed micelles during the transport within the gastrointestinal tract. Once they reach the intestinal wall, they can be absorbed integrally by intestinal epithelial cells due to the effect of endocytosis hence the oral absorption could be facilitated [41].

### 3.9. Pharmacokinetic Study

**Figure 9:**
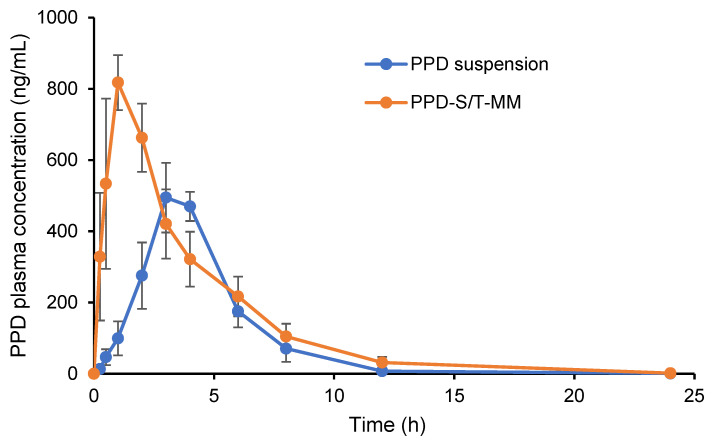
Mean plasma concentration-time profiles of PPD after intragastric administration of PPD-S/T-MM or PPD suspension in rats (mean ± SD, *n* = 3).

The plasma levels of PPD after intragastric administration of PPD suspension and PPD-S/T-MM formulation are shown in Figure 9, and the pharmacokinetic parameters including maximum plasma concentration (C_max_), time to reach the maximum plasma concentration (T_max_), area under the blood concentration-time curve (AUC) and half-life (T_1/2_) were calculated by WinnonLin software (Table 6). The results show that the plasma concentration of PPD increased rapidly after oral administration of PPD-S/T-MM (T_max_, 0.83 ± 0.29 h; C_max_, 844.33 ± 93.73 ng/mL). In comparison, oral administration of PPD suspension resulted in a longer T_max_ (3.33 ± 0.58 h) and lower C_max_ (531.33 ± 77.10 ng/mL). The AUC_0_–_24 h_ value for PPD-S/T/MM was approximately 1.58 times higher than that of PPD suspension. The results suggest that PPD-S/T-MM not only enables a shorter period to reach T_max_, which could clearly benefit to the fast management of acute schizophrenia but also enables higher oral bioavailability of PPD, which could reasonably extrapolate that superior PPD levels can be achieved in the brain after oral administration of PPD-S/T/MM over PPD suspension. The superior in vivo performance of PPD-S/T/MM should be attributed to the following reasons: (1) protection of the loaded PPD from crystallization in the gastrointestinal tract, (2) release of the PPD in a controlled manner at the wall of the small intestine [11], (3) prolongation of the residence time of the dissolved PPD in the gut due to the anti-nucleant performance of Soluplus^®^ [42], and (4) inhibition of efflux pumps by TPGS to improve the drug accumulation [43].

## 4. Conclusions

In this study, in order to improve the oral bioavailability of PPD, PPD-loaded Soluplus^®^/TPGS mixed micelles were successfully developed by the thin-film hydration method. The micelles were not only able to increase the aqueous solubility of PPD but also presented acceptable physical parameters and high encapsulation efficiency. The micelles were proved stable when they were diluted with water or when the environmental pH was changed. PPD was incorporated in the core of the mixed micelles as amorphous dispersion or solid solution, which was confirmed by DSC and XRD. The protection effect provided by the micellar structure retarded fast dissolution of PPD in SGF but enabled faster release in neutral solution in a controlled manner, compared with PPD release from its raw materials. PPD-S/T-MM successfully resulted in notably higher C_max_ and AUC than those of PPD suspension; meanwhile, it provided a faster rate to attain the C_max_. In conclusion, the use of the Soluplus^®^/TPGS mixed micellar system is able to increase the oral bioavailability of PPD in rats, suggesting the potential translation of the novel preclinical PPD oral formulation to clinical application for more effective management of acute schizophrenia.

## Figures and Tables

**Table 1:** PPD formulations approved by the U.S. FDA.

Brand Name	Active Ingredient	Dosage Form	Route of Administration	Strength	Manufacturer
Invega^®^	Paliperidone	Extended-release tablets	Oral administration	1.5 mg, 3 mg, 6 mg, 9 mg	JANSSEN PHARMS
Invega Sustenna^®^	Paliperidone palmitate	Long-acting injectables	Intramuscular injection	156 mg/mL	JANSSEN PHARMS
Invega Trinza^®^	Paliperidone palmitate	Long-acting injectables	Intramuscular injection	312 mg/mL	JANSSEN PHARMS

**Table 2:** Particle size, PDI and zeta potential of blank S/T-MM and PPD-S/T-MM (Mean ± SD, *n* = 3).

Types of the Mixed Micelles	Particle Size (nm)	PDI	Zeta Potential (mV)
Blank S/T-MM	61.3 ± 0.3	0.06 ± 0.02	−(1.65 ± 1.97)
PPD-S/T-MM-0.4	61.5 ± 0.7	0.05 ± 0.01	−(1.73 ± 4.63)
PPD-S/T-MM-1.0	61.3 ± 0.8	0.05 ± 0.01	−(2.89 ± 2.50)
PPD-S/T-MM-2.0	61.8 ± 0.9	0.04 ± 0.00	−(4.57 ± 1.64)

**Table 3:** Drug loading and encapsulation efficiency of PPD-S/T-MM (Mean ± SD, *n* = 3).

	Types of the Mixed Micelles		
	PPD-S/T-MM-0.4	PPD-S/T-MM-1.0	PPD-S/T-MM-2.0
DL (%)	0.78 ± 0.05	1.92 ± 0.01	3.69 ± 0.72
EE (%)	98.29 ± 1.67	97.41 ± 0.66	94.69 ± 2.19

**Table 4:** Particle size and PDI of PPD-S/T-MM in dilution stability study (Mean ± SD, *n* = 3).

Types of the Mixed Micelles	Dilution	Particle Size (nm)	PDI
PPD-S/T-MM-1.0	5-fold	61.34 ± 0.76	0.05 ± 0.01
	50-fold	62.44 ± 0.48	0.06 ± 0.01
	100-fold	62.89 ± 0.22	0.06 ± 0.02
PPD-S/T-MM-2.0	5-fold	61.77 ± 0.88	0.04 ± 0.00
	50-fold	61.12 ± 0.31	0.03 ± 0.02
	100-fold	62.93 ± 0.39	0.02 ± 0.01

**Table 5:** Particle size of PPD-S/T-MM in SGF and SIF (nm, Mean ± SD, *n* = 3).

Types of The Mixed Micelles	Duration of Exposure in SGF (h)			Duration of Exposure in SIF (h)		
	0	2	4	0	2	4
PPD-S/T-MM-1.0	61.2 ± 0.8	60.8 ± 1.5	58.3 ± 2.2	65.7 ± 0.8	64.2 ± 1.1	63.5 ± 0.4
PPD-S/T-MM-2.0	64.2 ± 1.1	62.7 ± 2.7	57.4 ± 1.4	67.2 ± 0.7	64.5 ± 1.1	63.6 ± 2.1

**Table 6:** Pharmacokinetic parameters of PPD after intragastric administration of PPD-S/T-MM or PPD suspension in rats (mean ± SD, *n* = 3).

Parameters	PPD-S/T-MM	PPD Suspension
T_max_ (h)	0.83 ± 0.29 *	3.33 ± 0.58
C_max_ (ng/mL)	844.33 ± 93.73 *	531.33 ± 77.10
T_1/2_ (h)	2.47 ± 0.20	2.08 ± 0.16
AUC_0→24 h_ (ng/mL·h)	3467.33 ± 705.33 *	2191.88 ± 607.99
AUC_0→∞_ (ng/mL·h)	3472.29 ± 709.18 *	2193.95 ± 609.60

* *p* < 0.05, compared with PPD suspension.

## Data Availability

All data available are reported in the article.

## References

[1] World Health Organization (WHO) Schizophrenia. https://www.who.int/news-room/fact-sheets/detail/schizophrenia.

[2] Xiang Y.T., Weng Y.Z., Leung C.M., Tang W.K., Ungvari G.S. (2008). Subjective quality of life in outpatients with schizophrenia in Hong Kong and Beijing: Relationship to socio-demographic and clinical factors. Qual. Life Res..

[3] Hatta K. (2015). Practical pharmacotherapy for acute schizophrenia patients. Psychiatry Clin. Neurosci..

[4] Välimäki M., Lantta T., Hätönen H.M., Kontio R., Zhang S.Y. (2016). Risk assessment for aggressive behaviour in schizophrenia. Cochrane Database Syst. Rev..

[5] Falkai P., Vogeley K. (2000). The chances of new atypical substances. Fortschr. Neurol. Psychiatr..

[6] Salarvand M., Ramezani V., Salarvand F., Darabi Z.A., Akrami M. (2021). Improvement of drug delivery properties of risperidone via preparation of fast dissolution tablet containing nanostructured microparticles. Iran. J. Pharm. Res..

[7] Al-Dhubiab B.E. (2017). Aripiprazole nanocrystal impregnated buccoadhesive films for schizophrenia. J. Nanosci. Nanotechnol..

[8] Pidaparthi K., Suares D. (2017). Comparison of nanoemulsion and aqueous micelle systems of paliperidone for intranasal delivery. AAPS Pharm. Sci. Tech..

[9] Sobczyński J., Chudzik-Rząd B., Grumezescu A.M. (2018). Mixed micelles as drug delivery nanocarriers. Design and Development of New Nanocarriers.

[10] Lasic D.D. (1992). Mixed micelles in drug delivery. Nature.

[11] Xu W., Ling P.X., Zhang T.M. (2013). Polymeric micelles, a promising drug delivery system to enhance bioavailability of poorly water-soluble drugs. J. Drug Deliv..

[12] USA Food and Drug Administration (FDA) Approval Package for NDA application of Invega Extended Release. https://www.accessdata.fda.gov/drugsatfda_docs/nda/2009/021999Orig1s004.pdf.

[13] Janicak P.G., Winans E.A. (2007). Paliperidone ER: A review of the clinical trial data. Neuropsychiatr. Dis. Treat..

[14] Tang Y.Y., Teng H., Shi Y.N., He H.B., Zhang Y., Yin T., Cai C.F., Tang X. (2018). Tablets of paliperidone using compression-coated technology for controlled ascending release. Asian J. Pharm. Sci..

[15] Piazzini V., Landucci E., Urru M., Chiarugi A., Pellegrini Giampietro D.E., Bilia A.R., Bergonzi M.C. (2020). Enhanced dissolution, permeation and oral bioavailability of aripiprazole mixed micelles: In vitro and in vivo evaluation. Int. J. Pharm..

[16] Ji S.P., Lin X., Yu E.J., Dian C.Y., Yan X., Li L.Y., Zhang M.M., Zhao W.C., Dian L.H. (2018). Curcumin-loaded mixed micelles: Preparation, characterization, and in vitro antitumor activity. J. Nanotechnol..

[17] Hu M., Zhang J.J., Ding R., Fu Y., Gong T., Zhang Z.R. (2017). Improved oral bioavailability and therapeutic efficacy of dabigatran etexilate via Soluplus-TPGS binary mixed micelles system. Drug Dev. Ind. Pharm..

[18] Sherje A., Londhe V. (2014). Inclusion complexes of hydroxy propyl-β-cyclodextrin and paliperidone: Preparation and characterization. Curr. Drug Discov. Technol..

[19] Wang Y.T., Ding Y.F., Xu Y.W., Wang C.Y., Ding Y.Y., Gao M., Ma C.G., Ma X.D., Li L. (2020). Mixed micelles of TPGS and Soluplus^®^ for co-delivery of paclitaxel and fenretinide: In vitro and in vivo anticancer study. Pharm. Dev. Technol..

[20] USA Food and Drug Administration (FDA) Dissolution Methods. Paliperidone. https://www.accessdata.fda.gov/scripts/cder/dissolution/dsp_SearchResults.cfm.

[21] Giri K., Lau M., Kuschnerus I., Moroni I., Garcia-Bennett A.E. (2020). A lysozyme corona complex for the controlled pharmacokinetic release of probucol from mesoporous silica particles. Biomater. Sci..

[22] Zhao Y.L., Cui Y.N., Li Y.M., Li L.B. (2014). Stable phosphatidylcholine-bile salt mixed micelles enhance oral absorption of paclitaxel: Preparation and mechanism in rats. J. Drug Target..

[23] Shen H.X., He D.D., Wang S.X., Ding P.G., Wang J.N., Ju J.M. (2018). Preparation, characterization, and pharmacokinetics study of a novel genistein-loaded mixed micelles system. Drug Dev. Ind. Pharm..

[24] Aravagiri M., Marder S.R. (2002). Brain, plasma and tissue pharmacokinetics of risperidone and 9-hydroxyrisperidone after separate oral administration to rats. Psychopharmacology.

[25] Bindu K.H., Reddy I.U., Anjaneyulu Y., Suryanarayana M.V. (2012). A stability-indicating ultra-performance liquid chromatographic method for estimation of related substances and degradants in paliperidone active pharmaceutical ingredient and its pharmaceutical dosage forms. J. Chromatogr. Sci..

[26] Stopková L., Gališinová J., Šuchtová Z., Čižmárik J., Andriamainty F. (2018). Determination of critical micellar concentration of homologous 2-alkoxyphenylcarbamoyloxyethyl-morpholinium chlorides. Molecules.

[27] BASF Corporation Technical Information—Soluplus^®^. https://pharma.basf.com/technicalinformation/30446233/soluplus.

[28] Bernabeu E., Gonzalez L., Cagel M., Gergic E.P., Moretton M.A., Chiappetta D.A. (2016). Novel Soluplus^®^—TPGS mixed micelles for encapsulation of paclitaxel with enhanced in vitro cytotoxicity on breast and ovarian cancer cell lines. Colloids Surf. B Biointerfaces.

[29] Alopaeus J.F., Hagesæther E., Tho I. (2019). Micellisation mechanism and behaviour of Soluplus^®^–furosemide micelles: Preformulation studies of an oral nanocarrier-based system. Psychopharmacology.

[30] Gaucher G., Satturwar P., Jones M.C., Furtos A., Leroux J.C. (2010). Polymeric micelles for oral drug delivery. Eur. J. Pharm. Biopharm..

[31] Jones M.C., Ranger M., Leroux J.C. (2003). pH-sensitive unimolecular polymeric micelles: Synthesis of a novel drug carrier. Bioconjug. Chem..

[32] Feng X., Chen Y., Li L., Zhang Y., Zhang L., Zhang Z. (2020). Preparation, evaluation and metabolites study in rats of novel amentoflavone-loaded TPGS/soluplus mixed nanomicelles. Drug Deliv..

[33] Zhao J., Xu Y.W., Wang C.Y., Ding Y.F., Chen M.Y., Wang Y.F., Peng J.Y., Li L., Lv L. (2017). Soluplus/TPGS mixed micelles for dioscin delivery in cancer therapy. Drug Dev. Ind. Pharm..

[34] Ding Y.Y., Wang C.Y., Wang Y.T., Xu Y.W., Zhao J., Gao M., Ding Y.F., Peng J.Y., Li L. (2018). Development and evaluation of a novel drug delivery: Soluplus^®^/TPGS mixed micelles loaded with piperine in vitro and in vivo. Drug Dev. Ind. Pharm..

[35] USA Food and Drug Administration (FDA) Prescribing Information for INVEGA^®^ (Paliperidone) Extended-Release Tablets. https://www.accessdata.fda.gov/drugsatfda_docs/label/2010/021999s018lbl.pdf.

[36] Kumar S., Randhawa J.K. (2013). Preparation and characterization of paliperidone loaded solid lipid nanoparticles. Colloids Surf. B Biointerfaces..

[37] Altamimi M.A., Neau S.H. (2017). Investigation of the in vitro performance difference of drug-Soluplus^®^ and drug-PEG 6000 dispersions when prepared using spray drying or lyophilization. Saudi Pharm. J..

[38] Wijiani N., Isadiartuti D., Rijal M.A.S., Yusuf H. (2020). Characterization and dissolution study of micellar curcumin-spray dried powder for oral delivery. Int. J. Nanomed..

[39] Hattori Y., Haruna Y., Otsuka M. (2013). Dissolution process analysis using model-free Noyes-Whitney integral equation. Colloids Surf. B Biointerfaces.

[40] Lu Y., Zhang E., Yang J.H., Cao Z.Q. (2018). Strategies to improve micelle stability for drug delivery. Nano Res..

[41] Wang J., Ma W., Tu P. (2015). The mechanism of self-assembled mixed micelles in improving curcumin oral absorption: In vitro and in vivo. Colloids Surf. B Biointerfaces..

[42] Zhong Y., Jing G.H., Tian B., Huang H., Zhang Y.Y., Gou J.X., Tang X., He H.B., Wang Y.J. (2016). Supersaturation induced by itraconazole/Soluplus^®^ micelles provided high GI absorption in vivo. Asian J. Pharm. Sci..

[43] Collnot E.M., Baldes C., Wempe M.F., Kappl R., Hüttermann J., Hyatt J.A., Edgar K.J., Schaefer U.F., Lehr C.M. (2007). Mechanism of inhibition of P-glycoprotein mediated efflux by vitamin E TPGS: Influence on ATPase activity and membrane fluidity. Mol. Pharm..

